# Recreation Bureau Notes

**Published:** 1893-07

**Authors:** 


### RECREATION BUREAU NOTES.

Chautauqua Lake, New York, is the Mecca of many a pilgrim in summer, who there finds rest and recreation, but never until now has it been regarded as a winter resort. The transformation of Sterlingworth Inn, the most fashionable and popular of all the famous summer hotels of Chautauqua, Saratoga (Lakewood), into a sanitarium for treatment of lung, throat and nervous diseases is therefore the more notable. The elegant surroundings and homelike comforts which have attracted thousands in summers past are rapidly filling the Inn, inside of which the potted plants and trees, combined with the warmth and verdure of the South, make one feel as though he had been transported to sunny Florida, or the balmy Bermudas, and while guest or patient, as the case may be, he feels as though his lines were cast in pleasant places and the quiet restfulness appeals to his sense of home comforts.

Rev. Joseph Newton Hallock, editor of the *Christian at Work*, writes after a return from this famous place:—It was not on account of the wonderful nature of Chautauqua Lake that we visited the locality. Nor was it entirely on account of its healthful influences that we left the busy city of New York for Lakewood. A report had come to our ears of an alleged discovery and so-called positive cure for consumption at the Sterlingworth Sanitarium. The much vaunted cures of alcoholism and the morphine habit taxed our credulity and we were skeptical about their lasting benefits; but the cure for consumption was something we positively disbelieved. But so we thought once of diphtheria, hydrophobia and Bright's disease, and hence when we were courteously invited to visit Sterlingworth and examine for ourselves, we promptly accepted the invitation. This Sanitarium is known to a large number of our readers from the fact that it was formerly an immense Inn; much of the great structure is still used as such during the summer months. The buildings themselves reminds us of the old castles in the middle ages, with all modern improvements.

We were received into the institution and duly introduced to the various physicians in charge of the establishment. In addition to a staff of three or four strictly regular physicians, there is a corps of well trained nurses to attend to all cases and watch every detail.

Two stories out of the great building are devoted exclusively to consumptives and the air in these is kept exactly at the temperature prescribed. These two stories were perfumed or "medicated" with something which reminded us of pine forest, and a long row of patients were inhaling some vapory substance from atomizers. But the physicians disclaimed the use of the atomizers, as of any essential or vital importance. They simply made use of it to assist nature in purifying the lungs and bronchial passages.

The real cure, as they asserted consisted in, first, the destruction of the bacilli and their pores, by the production and maintenance of a change in their environment inconsistent with their continued existence; secondly, in the prevention of sepsis and septic absorption by strictest attention to the most rigid principles of anti-sepsis, atmospheric, respiratory and gastro-intestinal; thirdly, by the promotion of the digestive, excretory and eliminative functions; and lastly by tonic and restorative treatment.

"Listerine," prepared by the Lambert Pharmacal Co., St. Louis, Mo., is a wonderful discovery. Its value in the treatment of Choleraic Diarrhœa, calls for attention just now, especially when fears are entertained that the dreaded disease will undoubtedly find its way again to this country.

"In time of peace prepare for war," is an old valued saying, and now that we have a discovery that will check the premonitory symptoms of the disease, look into its merits and be convinced. Listerine is not only valuable in this particular disease, but it is largely used in general practice by the physician, and the dentists pronounce it invaluable.

To tone up the system and rid yourself of any appearance of scrofula, catarrh, liver and kidney troubles, Murray's Infallible System Tonic is very strongly recommended by people who have learned its merits.

New baking powders seem to still come into existence, but there is none that the housewife so well likes and one that always bears a strong record, simply through its absolute purity, as the Royal. Use no other.

Have you tried the Australian Pill? Dr. Worst's liberal and generous offer to the readers of the Journal still holds good. If you have any liver, kidney, catarrh, or sick head-aches, it would be wise for you to communicate with him.

Van Houten's Cocoa, suggests to one who is addicted to a bilious or nervous temperament, not to use coffee, and it claims for itself, and well it may, a most delicious substitute. Give it a trial.

Tonics are always in order, for the system is seldom out of the line of needing something to " tone it up." There is none that equals Horsford's Acid Phosphate.

The St. Louis Hygienic College of Physicians and Surgeons, makes its annual announcement in this issue of the Journal. The college is open for men and women and it ranks among the best. Address S. W. Dodds, M. D., Dean, 2826 Washington Ave., St. Louis, Mo., for circulars and information.

Round Island, among the Thousand Islands, is one of the prettiest spots on the St. Lawrence River.

The Frontenac is situated in the centre of this island, which is one mile in length with walks, extending through and around the coast to the cottages which are occupied by the owners ; the healthful and exhilarating qualities of the atmosphere are unquestionable.

The Windsor Hotel, at Saratoga, is a popular hostelry to all who visit this famous summer resort.

Its cuisine is unequalled and its guests are always well cared for.

If you want good, wholesome mountain air at an elevation of two hundred and fifty feet above the beautiful and picturesque Hudson, visit " The Elmer," at Cornwall. The hotel is supplied with all modern conveniences.

The general topic of the day, and especially just now, vacation season, is the World's Fair. These questions come up in connection with the thought of making a visit to the "Windy City."

How shall I go? and where shall I stop? The latter we can answer for you. Stop at "The Linden," a beautiful hostelry located at 6504 Myrtle avenue, and but ten or fifteen minutes' walk from the Exposition grounds. The rates are very moderate, indeed. Write us for our handsome souvenirs of the hotel, or E. W. Mason, the proprietor.

For a thorough and complete education in Music, Elocution, Languages and the Fine Arts, there is no institution in the country that offers better inducements or advantages than the New England Conservatory of Music, located at Boston, Mass.

Instruction is given by seventy of the ablest American and European artists in all the advertised departments ; you are cordially invited to send to our Bureau for circulars of this institution and they will be promptly sent.

One of the best and at present very popular Sanitariums in this section of the country where salt water baths can be enjoyed for the promotion of health, is the new institution at Warsaw, Wyoming Co., New York. The baths alone are attaining great popularity among those afflicted with rheumatic and nervous troubles, and are considered by experts on the same level as the celebrated baths located at Nantwich, England, and Kreuznach, Germany, as to medicinal properties. The Sanitarium buildings are all new and supplied with all modern conveniences.

